# Fourier-Transform Infrared Spectroscopy as a Discriminatory Tool for Myotonic Dystrophy Type 1 Metabolism: A Pilot Study

**DOI:** 10.3390/ijerph18073800

**Published:** 2021-04-06

**Authors:** Tiago Mateus, Idália Almeida, Adriana Costa, Diana Viegas, Sandra Magalhães, Filipa Martins, Maria Teresa Herdeiro, Odete A. B. da Cruz e Silva, Carla Fraga, Ivânia Alves, Alexandra Nunes, Sandra Rebelo

**Affiliations:** 1 Department of Medical Sciences, Institute of Biomedicine (iBiMED), University of Aveiro, 3810-193 Aveiro, Portugal; tiagodcmateus@gmail.com (T.M.); idalia24@ua.pt (I.A.); adriana.e.costa@ua.pt (A.C.); dianaviegas97@ua.pt (D.V.); sandra.vicencia@ua.pt (S.M.); samartins@ua.pt (F.M.); teresaherdeiro@ua.pt (M.T.H.); odetecs@ua.pt (O.A.B.d.C.eS.); alexandranunes@ua.pt (A.N.); 2 Department of Chemistry, Aveiro Institute of Materials (CICECO), University of Aveiro, 3810-193 Aveiro, Portugal; 3 Neurology Department, Centro Hospitalar Tâmega e Sousa (CHTS), 4564-007 Penafiel, Portugal; cfraga@chts.min-saude.pt (C.F.); ivaniaalves83@gmail.com (I.A.)

**Keywords:** myotonic dystrophy type 1, Fourier-transform infrared spectroscopy, Principal Component Analysis, metabolomic profile, fibroblasts

## Abstract

Myotonic dystrophy type 1 (DM1) is a hereditary disease characterized by progressive distal muscle weakness and myotonia. Patients with DM1 have abnormal lipid metabolism and a high propensity to develop a metabolic syndrome in comparison to the general population. It follows that metabolome evaluation in these patients is crucial and may contribute to a better characterization and discrimination between DM1 disease phenotypes and severities. Several experimental approaches are possible to carry out such an analysis; among them is Fourier-transform infrared spectroscopy (FTIR) which evaluates metabolic profiles by categorizing samples through their biochemical composition. In this study, FTIR spectra were acquired and analyzed using multivariate analysis (Principal Component Analysis) using skin DM1 patient-derived fibroblasts and controls. The results obtained showed a clear discrimination between both DM1-derived fibroblasts with different CTG repeat length and with the age of disease onset; this was evident given the distinct metabolic profiles obtained for the two groups. Discrimination could be attributed mainly to the altered lipid metabolism and proteins in the 1800–1500 cm^−1^ region. These results suggest that FTIR spectroscopy is a valuable tool to discriminate both DM1-derived fibroblasts with different CTG length and age of onset and to study the metabolomic profile of patients with DM1.

## 1. Introduction

Myotonic dystrophy type 1 (DM1) is a hereditary multisystemic disease mainly characterized by progressive distal muscle weakness and myotonia (sustained muscle contractions) [1,2,3,4,5]. DM1 is caused by the expansion of unstable repetitions of the cytosine-thymine-guanine trinucleotide (CTG) in the 3’ untranslated region (3’UTR) of the *Myotonic Dystrophy Protein Kinase* (*DMPK*) gene located at chromosome 19q13.3 [4,5,6,7]. Due to DM1 clinical heterogeneity, disease severity can be subdivided according to symptoms severity and the age of onset [5,6,7,8]. In other words, DM1 is subdivided into three different phenotypes (mild, classic and severe) and clinically categorized as congenital-onset (<1 month and ≥1000 CTG repeat length), infantile-onset (1 month to 10 years and >500 CTG repeat length), juvenile-onset (10–20 years and >400 CTG repeat length), adult-onset (20–40 years and 150 to 1000 CTG repeat length) and late-onset (>40 years and 50 to 149 CTG repeat length) [6,7,8,9]. 

The frequency of Metabolic Syndrome (MetS) in patients with muscle disorders is significantly higher than in the general population [10,11] due to abnormal lipid metabolism particularly observed in skeletal muscle tissue of patients with DM1. The main causes of metabolic syndrome are insulin resistance, hypertriglyceridemia, increased fat mass, high levels of low-density lipoprotein (LDL), low levels of high-density lipoproteins (HDL), hypertension, and elevated levels of glucose and abdominal obesity [10,12,13,14,15,16,17]. In addition, it is interesting that patients with DM1 have a normal glucose tolerance and a very low prevalence of diabetes, in spite of a marked insulin resistance [17,18]. In a previous study, the significant difference between patients with DM1 and controls regarding the lipid metabolism was summarized. Briefly, patients with DM1 had increased insulin resistance by the homeostasis model assessment (HOMA) and elevated levels of triacylglycerol levels, total cholesterol, low-density lipoprotein and insulincompared to controls. However, high-density lipoproteins levels were lower than the controls [11]. Due to these features, patients with DM1 have a high propensity to develop a metabolic syndrome. Therefore, the evaluation of the metabolome of patients with DM1 represents an important step, since it provides rapid, sensitive and reproducible data to understand the differences between the observed DM1 phenotypes, allowing a better characterization and ideally distinguishing different phenotypes within patients with DM1. 

The study of the lipidome (lipidic metabolome) has evolved in the past years due to development of techniques such as Nuclear Magnetic Resonance (NMR), Mass Spectrometry (MS) and vibrational spectroscopy such as Raman and Fourier-transform infrared spectroscopy (FTIR) [19,20]. Although, FTIR is not as selective or sensitive as NMR and MS, it is able to identify the presence of carbohydrates, amino acids, lipids, and fatty acids, as well as proteins and polysaccharides, in the sample in one single experiment, recognized as a valuable tool for metabolic fingerprinting [19,21]. Infrared (IR) spectroscopy is a noninvasive and, in some cases, nondestructive type of technique based on the vibration of molecules’ covalent bonds as a result of the interaction of infrared radiation with a sample (tissues, body fluids or cells) [22,23,24]. Vibrational spectroscopy techniques are known as relatively simple and reproducible; spectra are obtained in a few minutes and spectroscopic bands are relatively narrow, easy to resolve, and sensitive to molecular structure, conformation, and environment [25]. Additionally, this requires a minimal sample preparation and small amount of biological material (from micrograms to nanograms) for analysis [25,26,27]. IR can be divided into three regions: near-IR region (13,000–4000 cm^−1^), mid-IR region (4000–400 cm^−1^), and far-IR region (<400 cm^−1^). Mid-IR region spectrum is currently considered the most promising spectroscopic technique for application in biomedical diagnostics [21,28] and can be divided into four regions, and the nature of a group frequency is related to the region in which it is located. These regions are defined as follows: X–H stretching region (4000–2500 cm^−1^), triple bond region (2500–2000 cm^−1^), double bond region (2000–1500 cm^−1^), and fingerprint region (1500–600 cm^−1^) [22]. 

One disadvantage of FTIR spectroscopy is the strong absorption of water in the mid-IR region. To overcome this disadvantage, experiments are carried out with dried samples [29]. A common method for biological sample analysis is the Attenuated Total reflectance FTIR spectroscopy (ATR-FTIR) [23,27], which is one of the approaches for FTIR Spectroscopy sampling that allows directly measuring the sample in the ATR crystal (crystal with a high refractive index) [30]. The ATR reduces the dispersion of the incident IR beam, increasing the sensitivity and reproducibility of the technique [26,30]. The resultant attenuated radiation is measured and plotted as a function of wavelength and gives rise to the absorption spectra characteristics of the sample [22,26,30].

FTIR spectroscopic data can be analyzed by direct spectra analyses or with the support of statistical tools (multivariate analysis). Multivariate analysis uses mathematical, statistical, and computer sciences to efficiently extract useful information from data generated [28]. One of the most used unsupervised methods is Principal Component Analysis (PCA), which does not require previous knowledge about the samples [28]. PCA reduces a large number of variables characteristic of a spectrum to a few variables or Principal Components that reflect the most relevant information and assist in resolving overlapping spectral features [28]. 

The contribution of the metabolic dysfunction for DM1 is not fully elucidated. Therefore, the aim of the present study is to verify if ATR-FTIR spectroscopy, together with multivariate analysis, is a suitable technique to provide a novel molecular insight into the characterization and distinction of the biochemical profile of patients with DM1 with different severities and different ages of disease onset and matched controls.

## 2. Material and Methods

### 2.1. DM1-Derived Fibroblast and Controls

DM1-derived fibroblasts and controls were obtained from two different sources, namely the Coriell Institute and our own research group, Neuroscience and Signaling Laboratory at University of Aveiro (Neurolab UA, Aveiro, Portugal).

DM1-derived fibroblasts and control from skin biopsies of donors were purchased from Coriell Institute. Five samples of fibroblasts were obtained and used: one was a control and four were diagnosed with DM1. Control-derived fibroblasts (GMO2673) comprise a CTG repeat length between 5 and 27. Two different DM1-derived fibroblasts with approximately 1000 CTG repeat length (GMO4033 and GMO4647) were also analyzed. Finally, two different DM1-derived fibroblasts with approximately 2000 CTG repeat length (GMO3989 and GMO3759) were also used. All donors were male and Caucasian with an age between 23 to 48 years. From this point, and all over the manuscript, the cell lines GMO4033 and GMO4647 will be denominated as DM1_1000 (1) and DM1_1000 (2), respectively. When analyzed together they will be denominated as DM1_1000. The same will happen with the cell lines GMO3759 and GMO3989, which will be denominated as DM1_2000 (1) and DM1_2000 (2), respectively. When analyzed together they were denominated as DM1_2000. Also, the cell line GMO2673 will be denominated as control. 

In addition, through a collaborative work developed at Neurolab UA and the Centro Hospitalar do Tâmega e Sousa, EPE (neurology department), approved by the Ethics Committee for Health of the Centro Hospitalar do Tâmega e Sousa, EPE (obtained at 14-08-2019 with approval number 31-2019), skin biopsies of adult patients with previous genetically confirmed DM1 and adult healthy volunteers without any neuromuscular disorder for the control group were collected. Moreover, the skin punch biopsies were collected from the patients with DM1 (with an age between 21 to 63 years) and from the control (with an age of 44 years) and skin-derived fibroblasts were established and cultured at Neurolab UA. For the present study 5 male DM1-derived fibroblasts and 1 control were chosen for ATR-FTIR spectroscopy analysis. These 5 DM1-derived fibroblasts were characterized by different ages of onset as congenital-onset (cDM1), infantile-onset (iDM1), juvenile-onset (jDM1), adult-onset (aDM1) and late-onset (lDM1).

### 2.2. Cell Culture

Fibroblasts purchased from Coriell Institute were cultured in complete Dulbecco’s Modified Eagle Medium, high glucose (DMEM; Gibco, Thermo Fisher Scientific, Waltham, MA, USA) supplemented with 15% Fetal Bovine Serum (FBS; Gibco, Origin South America) in T75 flasks. The culture medium was changed every two days. All washes were performed using Phosphate-buffered saline (PBS), without Ca^2+^ and Mg^2+^ (Thermo Fisher Scientific, Waltham, MA, USA). Whenever fibroblasts reached 80–90% confluence, they were subcultured using 0.05% trypsin-EDTA and transferred to new T75 flasks and maintained at 37 °C in a humidified 5% CO_2_ atmosphere.

Skin-derived fibroblasts were established and cultured in the Neurolab UA. Briefly, they were cultured in complete Dulbecco’s Modified Eagle Medium, high glucose (DMEM; Gibco, Thermo Fisher Scientific, Waltham, MA, USA) supplemented with 20% Fetal Bovine Serum (FBS; Gibco, Origin South America)and 1% penicillin/streptomycin in T75 flasks. The culture medium was changed every two days. All washes were performed using Phosphate-buffered saline (PBS), without Ca^2+^ and Mg^2+^ (Thermo Fisher Scientific, Waltham, MA, USA). Whenever fibroblasts reached 80–90% confluence, they were subcultured using 0.05% trypsin-EDTA and transferred to new T75 flasks and maintained at 37 °C in a humidified 5% CO_2_ atmosphere.

For FTIR spectroscopy analysis, a fibroblasts density of 0.9 × 10^6^ was used. Briefly, primary fibroblasts were cultured and after reaching around 90% of confluency (T75 flasks), the fibroblasts were PBS washed, trypsinazed using 0.05% trypsin-EDTA (Gibco solution and then resuspended in 3 mL of fresh complete medium and cell density determined as previously described [31,32]. A total of 0.9 × 10^6^ fibroblasts were used for each replicate.

### 2.3. FTIR Measurements

The DM1-derived fibroblasts and controls spectra were acquired using a FTIR spectrometer (Alpha Platinum ATR, Bruker Corporation, Billerica, MA, USA) equipped with a diamond ATR crystal, and preprocessed using OPUS software version 7.0 (Bruker Corporation, Billerica, MA, USA) Five µL of the DM1-derived fibroblasts and controls were placed in the crystal. To overcome the water interference, DM1-derived fibroblasts were air-dried before spectra were acquired. The spectra were obtained in the wavenumber range 4000–600 cm^−1^, with a resolution of 8 cm^−1^ and 64 co-added scans. Three replicates were obtained from DM1-derived fibroblasts and controls. Between different samples reading, the crystal was cleaned with 70% ethanol and distilled water and a background spectrum was acquired with the crystal empty (cleaned) to eliminate possible interference from fluctuations in the conditions of the room. All spectra acquisition was performed in a controlled room with a temperature of 23 °C and humidity of 35%. 

### 2.4. FTIR Data Analysis

All spectra were processed using The Unscrambler X software (v.10.4, CAMO Analytics, Oslo, Norway). All spectra were baseline corrected and area normalized. The normalized (duplicated matrix) spectra were derivatized, using the second derivate and Savitzky–Golay method with 3 smoothing points to resolve the overlapping peaks and maximize the differences between the spectra. Principal Component Analysis was applied to the normalized second-derivative spectra of all DM1-derived fibroblasts and controls. For multivariate analysis, three spectral region (mid-IR) 3000–2800 cm^−1^, 1800–1500 cm^−1^ and 1200–900 cm^−1^ were chosen, and all spectral assignments were made according to cited literature references [22,30,33,34]. In addition, the number of latent variables used for each PCA was considered and the percentage of information explained by each PC was taken into consideration.

### 2.5. Intensity Ratio Evaluation

In order to study possible disease-induced variation of the lipid content between control and DM1-derived fibroblasts with different CTG repeat length and the age of onset, the long hydrocarbon chains in lipids (CH_2_/CH_3_), lipid peroxidation (Carbonyl/Total lipid) and the unsaturated/saturated ratios were calculated by measuring peak intensity of the second derivative spectra [33,35]. Peak intensity for CH_2_ (~2922 cm^−1^—asymmetric vibration of the CH_2_ groups) and CH_3_ (~2959 cm^−1^—asymmetric vibration of the CH_3_) was used to calculate CH_2_ and CH_3_ ratio [35]. Peak intensity for C=O stretching (~1747 cm^−1^) and total lipid (CH_2_ sum of the saturated lipid bands (~2922 cm^−1^—asymmetric vibration of the CH_2_ groups and ~2851 cm^−1^—symmetric vibration of the CH_2_ groups)) was used to calculate Carbonyl/total lipid ratio [33,35]. Peak intensity for olefinic band (~3013 cm^−1^—olefinic band, C=CH stretching) and CH_2_ sum of the saturated lipid bands (~2922 cm^−1^—asymmetric vibration of the CH_2_ groups and ~2851 cm^−1^—symmetric vibration of the CH_2_ groups) was used to calculate unsaturated/saturated ratio [33,35].

Data were evaluated using a normality test, to decide whether parametric or nonparametric test should be used. To identify the differences in the lipid composition between groups, the ANOVA nonparametric statistical test was used, followed by the Dunnett’s test for multiple comparisons between groups. The data obtained were expressed as mean ± standard deviation, and the results were considered statistically significant for a *p* value ≤ 0.05 [35,36]. Also, the Mann–Whitney U nonparametric test was used to evaluate if the independent groups had significant differences and the Welch’s t-test was used to evaluate if there were any significant differences between the peak intensities from the different samples. The results obtained were expressed as mean ± standard deviation and were considered statistically significant for a *p* value ≤ 0.05. All statistical analysis was performed using GraphPad Prism v.6.01 (GraphPad Software, Inc., San Diego, CA, USA). 

In the first part of this manuscript, results of DM1-derived fibroblasts and control purchased from Coriell institute will be presented and, in the second part, the spectral and statistical analysis of DM1-derived fibroblasts and control from Neurolab UA will be shown. A comparison between the two data sets, using PCA, will also be presented. 

## 3. Results

### 3.1. FTIR Spectral Analysis of Fibroblasts from Coriell Institute

**Figure 1:**
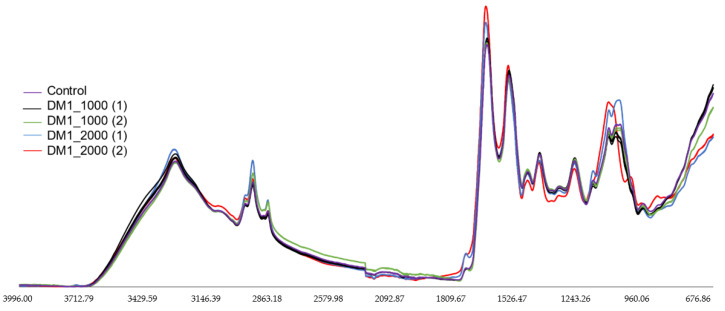
FTIR spectra of DM1-derived fibroblasts and control from Coriell Institute. Baseline-corrected and area normalized spectra of DM1-derived fibroblasts and control in the 4000–600 cm^−1^ region. *X*-axis:wavenumber cm^−1^, *Y*-axis: arbitrary units (AU).

In the current study, ATR-FTIR spectroscopy coupled with multivariate analysis (PCA) was performed to characterize and discriminate between DM1-derived fibroblasts with different CTG repeat length. Figure 1 shows ATR-FTIR spectral analysis in the region between 4000–600 cm^−1^ (Figure S1A). DM1-derived fibroblasts and control were analyzed in the 3000–2800 cm^−1^, 1800–1500 cm^−1^ and 1200–900 cm^−1^ spectral regions. In 3000–2800 cm^−1^ spectral region are located some of the bands assignments of lipids [30,33,34], namely vibrational asymmetric (υ_as_) and symmetric (υ_s_) stretching modes of –CH_3_ and CH_2_ [30,33,34] (Figure S1B). In the 1800–1500 cm^−1^ region are located bands associated mostly with proteins secondary structures, namely amide I band between 1700–1600 cm^−1^ and amide II band between 1600–1500 cm^−1^ region [27] and carbonyl band around 1747 cm^−1^ [22,27]. The amide I band is attributed to 80% of υ_s_(C=O), 10% N–H bending and 10% υ_s_(C–N). The amide II band is attributed to 60% of N–H bending and 40% υ_s_(C–N) [22,27,33] (Figure S1C). Additionally, in the region 1800–1500 cm^−1^, the band located around 1747–1736 cm^−1^ corresponds to an assignment of triacylglycerol, cholesterol esters and glycerophospholipids with a vibrational υ_s_(C=O) mode [27,30,33,34], which may be used to observe the degree of lipid peroxidation [36]. In the 1200–900 cm^−1^ region, the major bands are associated with carbohydrates and phosphates associated with nucleic acids [22,30,33] (Figure S1D).

### 3.2. Multivariate Analysis of the Spectroscopic Data of Fibroblasts from Coriell Institute

The first approach used in the present study was to assess whether this methodology can discriminate between DM1-derived fibroblasts and controls from the different groups (control, DM1_1000 (1 and 2) and DM1_2000 (1 and 2)) as well as to discriminate between DM1-derived fibroblasts (DM1_1000 (1 and 2) and DM1_2000 (1 and 2)) according to the increase of CTG length repeat. For that purpose, PCA analysis of the FTIR spectra was used. 

**Figure 2:**
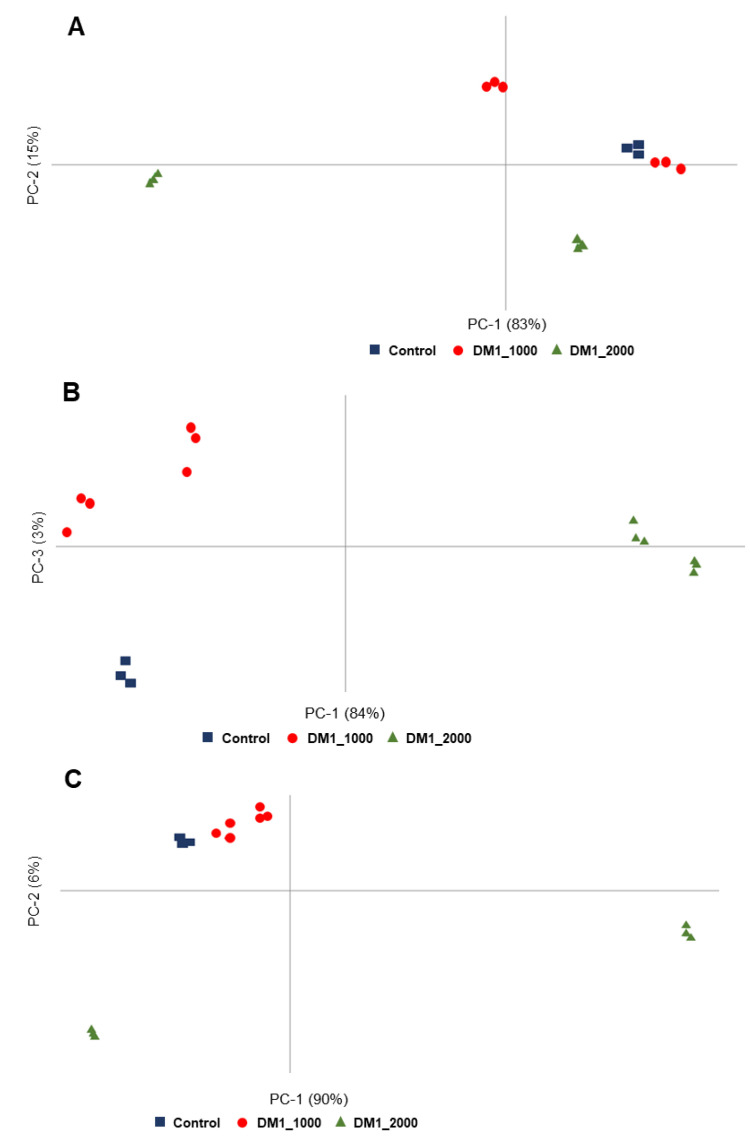
PCA scores profile of DM1-derived fibroblasts and control. (**A**) 3000–2800 cm^−1^ region. (**B**) 1800–1500 cm^−1^ region. (**C**) 1200–900 cm^−1^ region.

PCA results from the region 3000–2800 cm^−1^ are shown in Table 1 and illustrated in Figure 2A. In the PCA score it was possible to discriminate by principal component-2 (PC-2), where DM1_2000 was in the negative side of the PC-2 while control and DM1_1000 were in the positive side of PC-2 (Table 1), showing a clear separation between DM1_2000 and control and DM1_1000. According to the loading profile (Figure S2A), DM1_2000 was characterized by the spectroscopic signals located at 2953 cm^−1^ (CH_3_ asymmetric stretching), 2916 cm^−1^ (CH_2_ and CH_3_ stretching of phospholipids) and 2849 cm^−1^ (CH_2_ symmetric stretching), while control and DM1_1000 were characterized by the peaks 2925 cm^−1^ (CH_2_ asymmetric stretching) and 2874 cm^−1^ (CH_3_ symmetric stretching) (Figure S2A, Table 1).

The Figure 2B presents the PCA results from the region 1800–1500 cm^−1^. It was possible to discriminate between DM1-derived fibroblasts and controls by PC-1, where DM1_2000 was in the positive PC-1 separated from control and DM1_1000, which were in the negative PC-1 (Table 1). The loading plot showed that DM1_2000 was mainly characterized by the following spectral assignments 1628 cm^−1^ (Amide-I: parallel β-sheets (peptide, protein)), 1537 cm^−1^ (Amide II (proteins)) and 1509 cm^−1^ (CH_2_ bending (lipid, protein)), whereas the control and DM1_1000 was characterized by the following spectral assignments: 1747 cm^−1^ and 1736 cm^−1^ (C=O stretching (Triacylglycerol, cholesterol esters, glycerophospholipids)), 1696 cm^−1^ and 1682 cm^−1^ (Amide-I: antiparallel β-sheets (peptide, protein)), 1651 cm^−1^ (Amide-I: α-helices), 1554 cm^−1^ and 1523 cm^−1^ (Amide II (proteins)) (Figure S2B, Table 1). 

In addition, DM1_1000, located in Q2 (both negative PC-1 and positive PC-3) were discriminated from control located in Q3 (both negative PC-1 and PC-3) (Figure 2B). DM1_1000 was characterized by the following spectroscopic signals: 1693 cm^−1^ (Amide-I: antiparallel β-sheets (peptide, protein)) and 1639 cm^−1^ (Amide-I: parallel β-sheets (peptide, protein)) while the control was characterized by the following assignments 1747 cm^−1^ and 1743 cm^−1^ (C=O stretching (triacylglycerol, cholesterol esters, glycerophospholipids)), 1682 cm^−1^ (Amide-I: antiparallel β-sheets (peptide, protein)), 1651 cm^−1^ (Amide-I: α-helices), 1554 cm^−1^ and 1543 cm^−1^ (Amide II (proteins)) (Figure S2B, Table 1).

PCA results from the region 1200–900 cm^−1^ are shown in Table 1 and illustrated in Figure 2C. In the PCA scores it is possible to discriminate across PC-2 DM1_2000 from DM1_1000. DM1_2000 was located in the negative side of the PC-2, while together the control and DM1_1000 were located in the positive side of PC-2 (Table 1). PC-2 shows a clear discrimination between DM1_2000 and control and DM1_1000. According to the loading profile, DM1_2000 was characterized by the spectroscopic signals located at 1172 cm^−1^ (C–O stretching (Carbohydrates/glycogen, nucleic acids)), 1013 cm^−1^ (C–O stretching and C–OH bending (DNA and RNA, oligosaccharides, polysaccharides)), 991 cm^−1^ (C–O stretching (DNA and RNA ribose)) and 914 cm^−1^ (C–N^+^–C stretching (DNA and RNA ribose-phosphate chain vibration)), while together the control and DM1_1000 were characterized by the following spectroscopic signals: 1152 cm^−1^ (C–O stretching, C–O–H bending (Carbohydrates)), 1104 cm^−1^ and 1079 cm^−1^ (PO^2−^ symmetric stretching (DNA, RNA, phospholipid, phosphorylated protein)), 1053 cm^−1^ (C–O stretching and C–OH bending (DNA and RNA, oligosaccharides, polysaccharides)) and 968 cm^−1^ (PO_3_^2−^ stretching (DNA and RNA ribose)) 928 cm^−1^ (C–N^+^–C stretching (DNA and RNA ribose-phosphate chain vibration of RNA)) (Figure S2C, Table 1).

### 3.3. Intensity Ratios Obtained from Spectroscopic Data of Fibroblasts from Coriell Institute 

**Figure 3:**
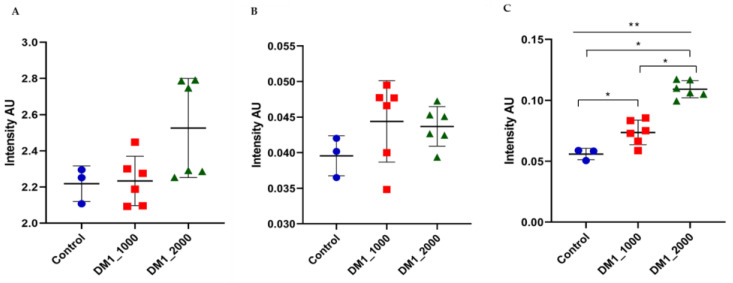
Scatter dot plot of DM1-derived fibroblasts and control ratios. (**A**) CH_2_/CH_3_ ratio (**B**) Unsaturated/Saturated ratios of (**C**) Carbonyl/Total lipid ratio. * *p* ≤ 0.05; ** *p* < 0.0001, Control (●); DM1_1000 (■); DM1_2000 (▲).

The CH_2_/CH_3_ ratio that translates the length of lipidic chains was not statistically significant between both DM1-derived fibroblasts (DM1_1000 and DM1_2000) and between the DM1-derived fibroblasts and the respective control. In addition, when applying the Kruskal–Wallis test (*p* = 0.1439) and Dunnett’s test there were not statistically significant differences (Figure 3A). The Unsaturated/Saturated ratio that represents the content of double bonds in the lipid structure was not statistically significant within the DM1-derived fibroblast and between the DM1-derived fibroblast and the control; there also was not a statistically significant difference when applying the Kruskal–Wallis test (*p* = 0.2571) and Dunnett’s (Figure 3B). The Carbonyl/Total lipid ratio that translates the carbonyl ester concentration in lipids showed significant different results between control and DM1_1000 (*p* = 0.0476), between control and DM1_2000 (*p* = 0.023), and between DM1_1000 and DM1_2000 (*p* = 0.0022). In addition, the Kruskal–Wallis test showed a significant result between the whole group with a *p* < 0.0001, and Dunnett’s test was also significant between control and DM1_2000 (Figure 3C).

Previous results regarding Coriell fibroblasts demonstrated a clear discrimination and statistical significance within the DM1-derived fibroblast (DM1_1000 and DM1_2000) and between DM1-derived fibroblasts and control, demonstrating the potential of FTIR technique as a discrimination tool. Thus, the same analytic procedure, PCA and intensity ratio evaluation, was applied to the spectroscopic data obtained from fibroblasts established at Neurolab UA, where the same experimental procedure was used, in order to validate the FTIR spectroscopy technique as a DM1 severities discriminatory tool. However, due to variables that we were not able to control related to the establishment of Coriell cell lines, the two data sets (DM1-derived fibroblasts from Coriell and from Neurolab) present completely distinct profiles and they were not possible to analyze together. The differences in the two data sets were confirmed by PCA results presented in (Figure S3). Interestingly, the cell lines established at Neurolab UA present very homogenous profiles when compared with ones from the Coriell Institute, which presented a high heterogeneity, not only between the Neurolab UA samples but also within the Coriell Institute samples, as seen in Figure S3.

### 3.4. FTIR Spectral Analysis of DM1-Derived Fibroblast from Neurolab UA

**Figure 4:**
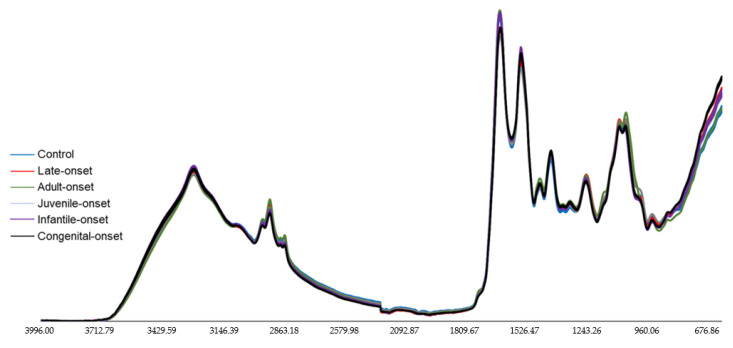
FTIR spectra of DM1-derived fibroblasts and control from Neurolab UA. Baseline-corrected and area normalized spectra of DM1-derived fibroblasts and control in the 4000–600 cm^−1^ region. *X*-axis:wavenumber cm^−1^, *Y*-axis: arbitrary units (AU).

ATR-FTIR spectroscopy and multivariate analysis (PCA) were performed to characterize and discriminate between DM1-derived fibroblasts according to the age of onset. Figure 4 shows ATR-FTIR spectral analysis in the region between 4000–600 cm^−1^ (Figure S4A). Briefly, the DM1-derived fibroblasts were analyzed in the 3000–2800 cm^−1^ (Figure S4B), 1800–1500 cm^−1^ (Figure S4C) and 1200–900 cm^−1^ (Figure S4D) regions, which were already discussed and explained previously. The same strategy was followed when analyzing the Neurolab UA control and DM1-derived fibroblasts. 

### 3.5. Multivariate Analysis of the Spectroscopic Data of Fibroblasts Cultured at Neurolab UA

The first approach was to assess whether the DM1-derived fibroblasts with different age of onset, namely late-onset, adult-onset, juvenile-onset, infantile-onset and congenital-onset, as well as the control, could be discriminated from each other through spectroscopic analysis and multivariate analysis. 

**Figure 5:**
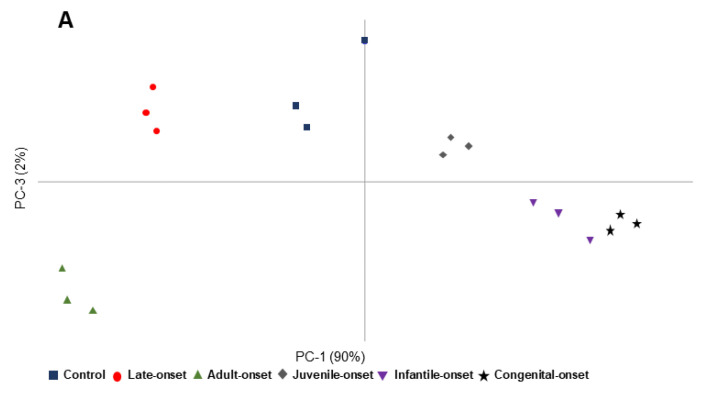

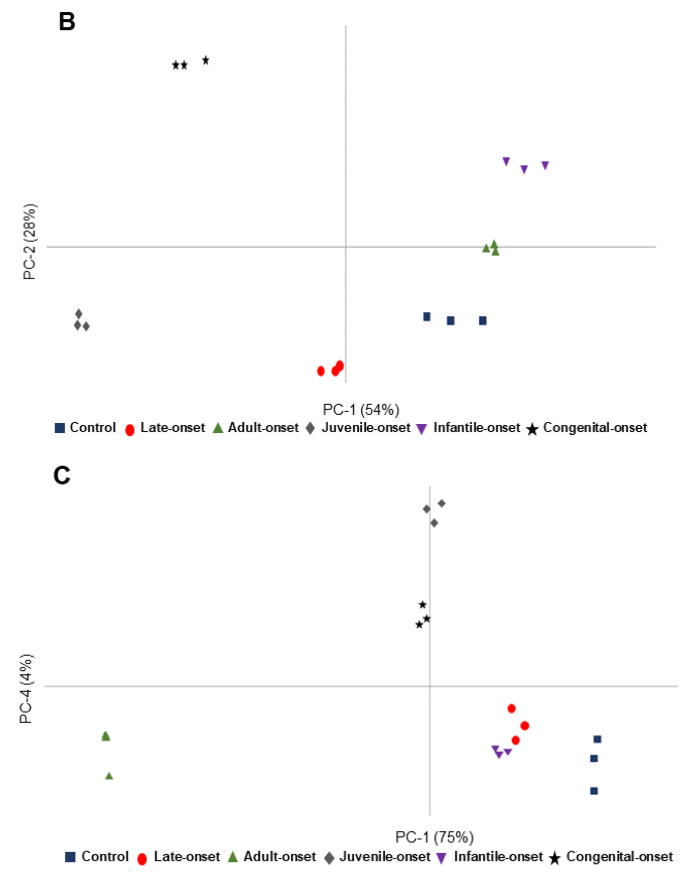
PCA scores profile of DM1-derived fibroblasts and control. (**A**) 3000–2800 cm^−1^ region. (**B**) 1800–1500 cm^−1^ region. (**C**) 1200–900 cm^−1^ region.

PCA results from 3000–2800 cm^−1^ are shown in Table 2 and illustrated in Figure 5A. Interestingly, using PCA score it was possible discriminate across PC-1, control, late-onset and adult-onset that are located in the PC-1 negative region and juvenile-, infantile- and congenital-onset that are located in the PC-1 positive region (Figure 5A). According to the loading profile plot, control, and late- and adult-onset were characterized by the spectroscopic signals at 2871 cm^−1^ (CH_3_ symmetric stretching), while juvenile-, infantile- and congenital-onset were characterized by the peaks 2959 cm^−1^ (CH_3_ asymmetric stretching), 2919 cm^−1^ (CH_2_ asymmetric stretching) and 2851 cm^−1^ (CH_2_ symmetric stretching) [30,34,36] (Table 2). In addition, infantile- and congenital-onset located at the Q4 were characterized by the following spectroscopic signals: 2956 cm^−1^ (CH_3_ asymmetric stretching), 2922 cm^−1^ (CH_2_ asymmetric stretching) and 2854 cm^−1^ (CH_2_ symmetric stretching) (Figure S5A, Table 2).

Figure 5B shows PCA results from 1800–1500 cm^−1^ regions (Table 2). In this case it was possible to discriminate the samples thought PC-2, where infantile- and congenital-onset were in the positive PC-2, and late-, adult- and juvenile-onset were located in the negative PC-2 (Figure 5B). The loading plot showed that infantile- and congenital-onset were characterized by 1747 cm^−1^ (C=O stretching), 1696 cm^−1^ and 1682 cm^−1^ (Amide-I: antiparallel β-sheets (peptide, proteins)), 1662 cm^−1^ (Amide-I: α-helices), 1639 cm^−1^ (Amide-I: parallel β- sheets (peptide, protein)) and 1523 cm^−1^ (Amide II (proteins)), while control, late-, adult- and juvenile-onset were characterized by the following spectral assignments 1648 cm^−1^ (Amide-I: α-helices), 1628 cm^−1^ (Amide-I: parallel β-sheets (peptide, protein)), 1551 cm^−1^ and 1537 cm^−1^ (Amide II (proteins)) and 1512 cm^−1^ (Lipid, protein) (Figure S5B, Table 2). 

In addition, congenital-onset could be discriminated from control and late-, adult-, juvenile-, and infantile-onset. In fact, infantile-onset was in the Q1 (positive PC-1 and negative PC-2), congenital-onset was in the Q2 (negative PC-1 and positive PC-2), late- and juvenile-onset were located at Q3 (negative PC-1 and PC-2), and control and adult-onset were located at Q4 (positive PC-1 and negative PC-2) (Figure 5B). Congenital-onset was characterized by: 1747 and 1736 cm^−1^ (C=O stretching), 1696 cm^−1^ and 1682 cm^−1^ (Amide-I: antiparallel β-sheets (peptide, protein)) and 1639 cm^−1^ (Amide-I: parallel β-sheets (peptide, protein)). Control and adult-onset were characterized by the peaks located at 1747 cm^−1^ and 1743 cm^−1^ (C=O stretching), 1682 cm^−1^ (Amide-I: antiparallel β-sheets (peptide, protein)), 1651 cm^−1^ (Amide-I: α-helices), 1554 cm^−1^ (Amide II (proteins)) and 1543 cm^−1^ (Amide II (proteins)) (Figure S5B, Table 2). 

PCA results from 1200–900 cm^−1^ region are shown in Table 2 and illustrated in Figure 5C. In the PCA score it is possible discriminate by PC-4, where juvenile- and congenital-onset were in the positive side of the PC-4 while control, late-, adult- and infantile-onset were in the negative side of PC-4 (Figure 5C). PC-4 allowed a clear separation between juvenile- and congenital-onset samples and control, late-, adult- and infantile-onset samples. According to the loading plot, juvenile- and congenital-onset samples were characterized by the spectroscopic signals located 1121 cm^−1^ (Phosphodiester groups of PO^2−^ (RNA)), 1084 cm^−1^ (PO^2−^ symmetrical stretching (DNA, RNA, phospholipid, phosphorylated protein)), 1047 cm^−1^ (C–O stretching and C–OH bending (DNA, RNA, oligosaccharides, polysaccharides)), 994 cm^−1^ (C–O stretching (DNA and RNA ribose)), 968 cm^−1^ (PO_3_^2−^ stretching (DNA and RNA ribose)) and 914 cm^−1^ (C–N^+^–C stretching (DNA and RNA ribose-phosphate chain vibration)), while control, late-, adult- and infantile-onset samples were characterized by the following spectroscopic signals 1155 cm^−1^ (C–O stretching, C–O–H bending (Carbohydrates)), 1076 cm^−1^ (PO^2−^ symmetrical stretching (DNA, RNA, phospholipid, phosphorylated protein)), 1025 cm^−1^ (C–O stretching and C–OH bending (DNA, RNA, oligosaccharides, polysaccharides)), 923 cm^−1^ (C–N^+^–C stretching (DNA and RNA ribose-phosphate chain vibration)) (Figure S5C, Table 2).

Also, control, late- and infantile-onset samples could be discriminated from adult-, juvenile- and congenital-onset samples. Juvenile-onset sample was in the Q1 (positive PC-1 and negative PC-2), congenital-onset was in the Q2 (negative PC-1 and positive PC-2), adult-onset sample was located at Q3 (negative PC-1 and PC-2), and control, late- and infantile-onset samples were located at Q4 (positive PC-1 and negative PC-2) (Figure 5C). Juvenile-onset sample was characterized by the peaks: 1124 cm^−1^ (Phosphodiester groups of PO^2−^ (RNA)) and 996 cm^−1^ (C–O stretching (DNA and RNA ribose)). Congenital-onset sample was characterized by the peak at 914 cm^−1^ (C–N^+^–C stretching (DNA and RNA ribose-phosphate chain vibration)). Adult-onset sample was characterized by the peak at 1112 cm^−1^ (Phosphodiester groups of PO^2−^ (RNA)). Control, late- and infantile-onset samples were characterized by the following peaks: 1155 cm^−1^ (C–O stretching, C–O–H bending (Carbohydrates)), 1079 cm^−1^ (PO^2−^ symmetrical stretching (DNA, RNA, phospholipid, phosphorylated protein)), 1042 cm^−1^ and 1022 cm^−1^ (C–O stretching and C–OH bending (DNA, RNA, oligosaccharides, polysaccharides)) and 926 cm^−1^ (C–N^+^–C stretching (DNA and RNA ribose-phosphate chain vibration)) (Figure S5C, Table 2). 

### 3.6. Intensity Ratios Obtained from Spectroscopic Data of DM1-Derived Fibroblasts Cultured at Neurolab UA

**Figure 6:**
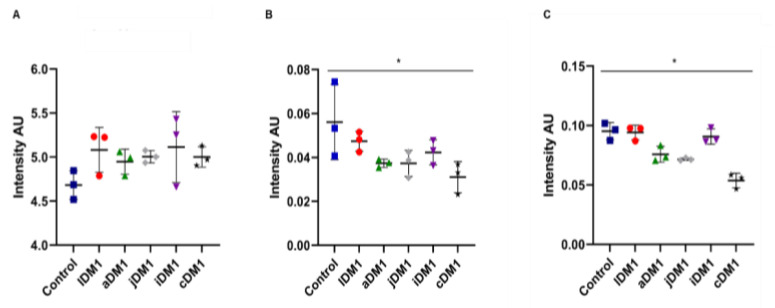
Scatter dot plot of DM1-derived fibroblasts and control ratios. (**A**) CH_2_/CH_3_ ratio (**B**) Unsaturated/Saturated ratios of (**C**) Carbonyl/Total lipid ratio. * *p* ≤ 0.05. AU—arbitrary units; Control (■); lDM1—late-onset DM1 (●); aDM1—adult-onset DM1 (▲) ; jDM1—juvenile-onset DM1 (◆); iDM1—infantile-onset DM1 (▼); cDM1—congenital-onset DM1 (★).

The CH_2_/CH_3_ ratio that translates the length of lipidic chains was not statistically significant between control and all DM1-derived fibroblasts tested and within DM1-derived fibroblast tested (late-, adult-, juvenile-, infantile- and congenital-onset); the Kruskal–Wallis test (*p* = 0.3538) and Dunnett’s test also were not statistically significant (Figure 6A). The Unsaturated/Saturated ratio that describes the content of double bonds in the lipid structure was not statistically significant between the control and DM1-derived fibroblasts tested and within the DM1-derived fibroblast (late-, adult-, juvenile-, infantile- and congenital-onset). Whereas when comparing the whole group using the Kruskal–Wallis test the results were statistically significant (*p* = 0.0459) as well as when Dunnett’s test was used (Figure 6B). The Carbonyl/Total lipid ratio that illustrates the carbonyl ester concentration in lipids was not statistically significant between the control and all DM1-derived fibroblasts tested and within the DM1-derived fibroblast (late-, adult-, juvenile-, infantile- and congenital-onset), whereas the Kruskal–Wallis test showed a statistically significant difference between the whole group (*p* = 0.0135) (Figure 6C). 

## 4. Discussion

Patients with DM1 present several metabolic alterations, namely insulin resistance, hypertriglyceridemia, increased fat mass, high levels of low-density lipoprotein (LDL), low levels of high-density lipoproteins (HDL), hypertension, and abdominal obesity [10,12,13,14,15,16,17]. Given these metabolic dysfunctions observed in patients with DM1, the evaluation of the metabolic profile of those patients is of extreme importance. For that purpose, in the present study, we performed ATR-FTIR spectroscopy analysis coupled with PCA (an exploratory tool of multivariate analysis) to characterize and discriminate the biomolecular profile within skin DM1-derived fibroblasts and a control established either at Coriell Institute or at Neurolab UA.

The analysis of the DM1-derived fibroblasts from Coriell Institute showed clear alterations between DM1-derived fibroblasts with different CTG repeat length (DM1_1000 with DM1_2000) at the molecular level, and the same happened with those established at Neurolab. These alterations were mainly observed in the 1800–1500 cm^−1^ region. In both spectral regions of 3000–2800 cm^−1^ and 1800–1500 cm^−1^ it was also, possible to discriminate between DM1-derived fibroblasts and control group using both fibroblasts groups (Coriell Institute and Neurolab UA).

In the DM1-derived fibroblasts (Coriell Institute, Camden, NJ, USA), there was a clear discrimination, provided by PCA analysis, between both DM1-derived fibroblasts and controls across PC-2. This discrimination is due to spectral assignments in the 3000–2800 cm^−1^ spectroscopic region in which DM1_2000 was characterized by a higher contribution of CH_3_ stretching signal, which may be associated with shorter lipidic stretching, while control and DM1_1000 were characterized by CH_2_ stretching signal, which can be associated with longer lipid chains [35,36].

Regarding the Neurolab UA DM1-derived fibroblasts, a clear discrimination between the most severe forms of DM1 (congenital-onset, infantile-onset and juvenile-onset) from control, late-onset and adult-onset was possible in the 3000–2800 cm^−1^ region and across PC-1. The most severe forms of DM1 were characterized by both CH_3_ and CH_2_ asymmetric stretching, meaning that these forms may have a higher number of lipid content in comparison to the control, late-onset and adult-onset form. This higher number of shorter lipidic stretching may be correlated with lipin 1 alterations previously described in DM1 [37,38]. Of note, lipin is a key enzyme that regulates transcriptional coactivators of fatty acid β-oxidation, lipid metabolism and signaling through the conversion of phosphatidic acid into diacylglycerol, in which diacylglycerol formations will be converted into triacylglycerol, phosphatidylethanolamine and/or phosphatidylcholine [11,39,40,41,42]. In a previous study, the common features of DM1 abnormal metabolic alterations and the potential role of lipin with dyslipidemia, insulin resistance, skeletal-muscle impairment, immune system (macrophages) and adipose tissues were summarized [11], demonstrating once again the importance of further studies related to the lipid metabolism of patients with DM1. In addition, present results could be associated with the increase of lipid peroxidation, consistent with the decrease in the olefinic content in the cellular membrane of DM1-derived fibroblasts (unsaturated/saturated ratio) due to the oxidation and degradation of lipids, since lipid peroxidation modifies the physical properties of lipids as demonstrated in previous studies, as described below [35,36].

In the spectral region of 1800–1500 cm^−1^, it was possible to discriminate DM1_2000 samples from control and DM1_1000 (Coriell Institute, Camden, NJ, USA), across PC-1. DM1_2000 samples were characterized by the peak assigned to Amide-I whose location may indicate the parallel β-sheet associated with protein aggregates [30,43]. Amide-I: parallel β-sheets are energetically less stable than Amide-I: antiparallel β-sheets and Amide-I: α-helices, and occur more rarely in proteins [44]. In addition, these protein aggregates may be related to the abnormal size of transcribed mutant RNA (CTG expansion). These mutant RNA with CUG repeats in the nucleus accumulates as a ribonuclear foci, sequestering and deregulating muscleblind-like protein1 (MBNL1) and CUG-BP-ELAV-Like family member1 (CUGBP1 or CELF1), which are important regulators of alternative splicing of other genes and core proteins in DM1 disease complexity [11,45,46]. Control and DM1_1000 were identified mainly by C=O stretching (Triacylglycerol, cholesterol esters, glycerophospholipids) and by Amide-I: antiparallel β-sheets and Amide-I: α-helices. Despite the differences in the spectral assignments of Amide I group, which may be due to the protein mixture of the secondary structure, the increase of C=O stretching band corresponds to the lipid peroxidation and seems to be the principal reason of discrimination between the control and DM1_1000 [35,36].

As for Neurolab DM1-derived fibroblast in the spectral region of 1800–1500 cm^−1^, it was possible to discriminate between the congenital and infantile-onset from the control, late-onset, adult-onset and juvenile-onset provided by PC-2. The congenital-onset and infantile-onset fibroblasts were mainly identified by the C=O stretching (Triacylglycerol, cholesterol esters, glycerophospholipids), which may be related to the lipid peroxidation. Lipid peroxidation is an oxidative degradation of lipids (e.g., membrane lipids, such as glycolipids, phospholipids and cholesterol), in which there is increased degradation of polyunsaturated fatty acids, giving rise to peroxides primary products, and they are then broken down into secondary products with shorter hydrocarbon chains and carbonyl compounds [35,36,47,48]. The increased lipid peroxidation may lead to atherosclerosis, Alzheimer’s disease, rheumatic arthritis, cancer, and immune disorders, among other features, since it causes uncontrolled oxidative stress, alterations in cell signaling, protein and DNA damage, and cytotoxicity [35,47,49]. 

In the 1200–900 cm^−1^, there is a clear discrimination of DM1_2000 from control and DM1_1000 through PC-2 analysis. However, it is difficult to infer biochemical differences between the samples since this region is characterized by many different biomolecules, namely fatty acids, sugars, amino acids, and nucleic acids, among others [23,25,33,35]. As for the spectral region of 1200–900 cm^−1^, there is a clear discrimination of congenital-onset and juvenile-onset from control group, late-onset, adult-onset and infantile-onset provided by PC-4. As described above, it is difficult to infer biochemical differences between the samples since this region is characterized by many different biomolecules [23,25,35,36].

The statistical analysis also showed significant results between DM1_2000 and control samples and DM1_1000 sample, in the 1800–1500 cm^−1^ region, once again showing the importance of the lipid peroxidation as a valuable target to discriminate between patients with different severities and to do further studies. In addition, when we applied the Welch’s t-test in the Coriell Institute samples, different peak intensities such as 2916 cm^−1^ and 2849 cm^−1^ from DM1_2000 were considered significantly different from the 2925 and 2874 peak from DM1_1000 and control samples in the 3000–2800 cm^−1^ region, and the same was observed in the 1200–900 cm^−1^ region where 1013 cm^−1^, 991 cm^−1^ and 914 cm^−1^ from DM1_2000 were considered significantly different from the 1152 cm^−1^ 1104 cm^−1^ and 1053 cm^−1^ from the DM1_1000 and control samples. Furthermore, the same was also observed only in the 3000–2800 cm^−1^ region in the Neurolab UA samples; namely, the peak intensities 2919 cm^−1^ and 2851 cm^−1^ from the congenital- infantile- and juvenile-onset samples were considered significantly different from the 2942 cm^−1^ and 2840 cm^−1^ from the adult-, late-onset and control samples.

Interestingly, changes in lipid absorption (3000–2800 cm^−1^ region) and ester cholesterol, phospholipids and proteins absorption (1800–1500 cm^−1^ region) occur in both cell groups (Coriell and Neurolab UA). However, the 1800–1500 cm^−1^ region clearly discriminates the most severe forms of DM1 disease (congenital-onset and infantile-onset) and DM1_2000 samples from the other forms of DM1 disease (late-onset, adult-onset and juvenile-onset) and DM1_1000 samples. Also, it was possible to discriminate between the most severe forms of DM1 disease and DM1_2000 samples from the control groups. These discriminations mean that there are changes in lipid content and in lipid peroxidation, and that ATR-FTIR spectroscopy analysis of 1800–1500 cm^−1^ region may be the region of choice to help discriminate between the different severity forms of DM1 disease. 

Although there were promising results, this study has some limitations, due to the small sample size; however, we should keep in mind that DM1 is a rare disease with high limitations concerning sample obtention. Regarding DM1-derived fibroblasts obtained from Coriell Institute, several variables are related to the protocol since collection until the culturing is unknown, which may be present on the observed in the present study (Figure S3). Therefore, it is important to establish a large cohort, increasing the Neurolab UA cohort, with more heterogeneity of the severities of the DM1 disease, to take into consideration all variabilities, meaning that a strict protocol must be followed, in which all variables are known, in order to obtain a consistent homogeneity of the samples. Also, it is important to statistically analyze the differences of the lipidic profile between the different severities DM1 disease. It would be interesting for future studies to analyze DM1-derived fibroblasts through other metabolic techniques such as NMR and MS to understand deeply which classes and species of lipids are responsible for changes in the metabolism profile of patients with DM1, since ATR-FTIR spectroscopy is incapable of detecting specific metabolites that are responsible for causing metabolic changes. 

## 5. Conclusions

The present work demonstrated that ATR-FTIR spectroscopy together with multivariate analysis (PCA) is a valuable and useful technique as a screening tool for DM1 patients’ discrimination according the CTG repeat length and the age of onset of clinical symptoms, particularly in the 1800–1500 cm^−1^ region. Further, it can give important biochemical insights regarding lipid metabolism alterations, allowing the understanding of the DM1 disease complexity and further research towards the development of DM1 therapeutics strategies.

The very promising results obtained, although with a small sample size, highlight even more the importance of FTIR spectroscopy technique as an innovative discrimination tool, never before used in patients with DM1, in such a fundamental domain as lipid metabolism analysis. However, further studies using more specific metabolic techniques, namely NMR and MS, should also be addressed in DM1.

## Figures and Tables

**Table 1:** DM1-derived fibroblast (Coriell Institute) and control spectroscopic signals, assignments and vibrational mode obtained by Principal Component Analysis (PCA) in the 3000–2800 cm^−1^, 1800–1500 cm^−1^ and 1200–900 cm^−1^ regions. Λ—Wavenumber; Q—Quadrant; −—Negative PC region; +—Positive PC region; DM1_2000 (1 and 2)—DM1_2000 (1) and DM1_2000 (2); DM1_1000 (1 and 2)—DM1_1000 (1) and DM1_1000 (2).

**3000–2800 cm^−1^ Region**			
**Discrimination across PC-2**	**λ (cm^−1^)**	**Vibrational Mode**	**Assignments**
PC-2 – DM1_2000 (1 and 2)	2953	CH_3_ asymmetric stretching	Lipid (long chain fatty acids, phospholipids)
	2916	CH_2_ and CH_3_ stretching of phospholipids	
	2849	CH_2_ symmetric stretching	
PC-2 + Control and DM1_1000 (1 and 2)	2925	CH_2_ asymmetric stretching	
	2874	CH_3_ symmetric stretching	
**1800–1500 cm^−1^ Region**			
**Discrimination across PC-1**	**λ (cm^−1^)**	**Vibrational Mode**	**Assignments**
PC-1 – Control and DM1_1000 (1 and 2)	1747	C=O stretching	Triacylglycerol, cholesterol esters, glycerophospholipids
	1736		
	1696	80% C=O stretching, 10% N–H bending, 10% C–N stretching	Amide-I: anti-parallel β-sheets (peptide, protein)
	1682		Amide-I: anti-parallel β- sheets (peptide, protein)
	1651		Amide-I: α- helices
	1554	60% N–H bending, 40% C–N stretching	Amide II (proteins)
	1523		
PC-1 + DM1_2000 (1 and 2)	1628	80% C=O stretching, 10% N–H bending, 10% C–N stretching	Amide-I: parallel β- sheets (peptide, protein)
	1537	60% N–H bending, 40% C–N stretching	Amide II (proteins)
	1509	CH_2_ bending	Lipid, protein
**1800–1500 cm^−1^ Region (Q2 and Q3)**			
**Quadrant-2**	**λ (cm^−1^)**	**Vibrational Mode**	**Assignments**
DM1_1000 (1 and 2)	1693	80% C=O stretching, 10% N–H bending, 10% C–N stretching	Amide-I: anti-parallel β-sheets (peptide, protein)
	1639		Amide-I: parallel β- sheets (peptide, protein)
Quadrant-3	λ (cm^−1^)	Vibrational mode	Assignments
Control samples	1747-1743	C=O stretching	Triacylglycerol, cholesterol esters, glycerophospholipids
	1682	80% C=O stretching, 10% N–H bending, 10% C–N stretching	Amide-I: anti-parallel β- sheets (peptide, protein)
	1651		Amide-I: α- helices
	1554	60% N–H bending, 40% C–N stretching	Amide II (proteins)
	1543		
**1200–900 cm^−1^ Region**			
**Discrimination across PC-2**	**λ (cm^−1^)**	**Vibrational Mode**	**Assignments**
PC-2 – DM1_2000 (1 and 2)	1172	C–O stretching	Carbohydrates/glycogen, nucleic acids
	1013	C–O stretching and C–OH bending	DNA and RNA, oligosaccharides, polysaccharides (e.g., glucose)
	991	C–O stretching	DNA and RNA ribose
	914	C–N^+^–C stretching	DNA and RNA ribose-phosphate chain vibration of RNA
PC-2 + Control and DM1_1000 (1 and 2)	1152	C–O stretching, C–O–H bending	Carbohydrates
	1104	PO^2−^ symmetrical stretching	DNA, RNA, phospholipid, phosphorylated protein
	1079		
	1053	C–O stretching and C–OH bending	DNA and RNA, oligosaccharides, polysaccharides (e.g., glucose)
	968	PO_3_^2−^ stretching	DNA and RNA ribose
	928	C–N^+^–C stretching	DNA and RNA ribose-phosphate chain vibration of RNA

**Table 2:** Neurolab UA DM1-derived fibroblast and control spectroscopic signals, assignments and vibrational mode obtained by PCA in the 3000–2800 cm^−1^, 1800–1500 cm^−1^ and 1200–900 cm^−1^ regions. λ—Wavenumber Q—Quadrant; −—Negative quadrants; +—Positive quadrants; DM1—derived fibroblast; lDM1—late-onset; aDM1—adult-onset; jDM1—juvenile-onset; iDM1—infantile-onset; cDM1—congenital-onset.

**3000–2800 cm^−1^ Region**			
**Discrimination across PC-1**	**λ (cm^−1^)**	**Vibrational Mode**	**Assignments**
PC-1 − Control, lDM1, aDM1	2871	CH_3_ symmetric stretching	Lipid (long chain fatty acids, phospholipids)
PC-1 + jDM1, iDM1 and cDM1	2959	CH_3_ asymmetric stretching	
	2919	CH_2_ asymmetric stretching	
	2851	CH_2_ symmetric stretching	
**3000–2800 cm^−1^ region (Q1, Q3 and Q4)**			
**Quadrant-1**	**λ (cm^−1^)**	**Vibrational Mode**	**Assignments**
jDM1	No peaks	NA	Lipid (long chain fatty acids, phospholipids)
Quadrant-3	λ (cm^−1^)	Vibrational mode	Assignments
aDM1	No peaks	NA	Lipid (long chain fatty acids, phospholipids)
Quadrant-4	λ (cm^−1^)	Vibrational mode	Assignments
iDM1 and cDM1	2956	CH_3_ asymmetric stretching	Lipid (long chain fatty acids, phospholipids)
	2922	CH_2_ asymmetric stretching	
	2854	CH_2_ symmetric stretching	
**Mid-IR bands at 1800–1500 cm^−1^ Region**			
**Discrimination across PC-2**	**λ (cm^−1^)**	**Vibrational Mode**	**Assignments**
PC-2 − Control, lDM1, aDM1 and jDM1	1648	80% C=O stretching, 10% N–H bending, 10% C–N stretching	Amide-I: α- helices
	1628	80% C=O stretching, 10% N–H bending, 10% C–N stretching	Amide-I: parallel β- sheets (peptide, protein)
	1551	60% N–H bending, 40% C–N stretching	Amide II (proteins)
	1537	60% N–H bending, 40% C–N stretching	Amide II (proteins)
	1512	CH_2_ bending	Lipid, protein
PC-2 + iDM1 and cDM1	1747	C=O stretching	Triacylglycerol, cholesterol esters, glycerophospholipids
	1696	80% C=O stretching, 10% N–H bending, 10% C–N stretching	Amide-I: anti-parallel β-sheets (peptide, protein)
	1682	80% C=O stretching, 10% N–H bending, 10% C–N stretching	Amide-I: anti-parallel β-sheets (peptide, protein)
	1662	80% C=O stretching, 10% N–H bending, 10% C–N stretching	Amide-I: α- helices
	1639	80% C=O stretching, 10% N–H bending, 10% C–N stretching	Amide-I: parallel β- sheets (peptide, protein)
	1523	60% N–H bending, 40% C–N stretching	Amide II (proteins)
**1800–1500 cm^−1^ (Q1, Q2, Q3 and Q4)**			
**Quadrant-1**	**λ (cm^−1^)**	**Vibrational Mode**	**Assignments**
iDM1	No peak	NA	NA
Quadrant-2	λ (cm^−1^)	Vibrational mode	Assignments
cDM1	1747	C=O stretching	Triacylglycerol, cholesterol esters, glycerophospholipids
	1736		
	1696	80% C=O stretching, 10% N–H bending, 10% C–N stretching	Amide-I: anti-parallel β-sheets (peptide, protein)
	1682		
	1639		Amide-I: parallel β- sheets (peptide, protein)
Quadrant-3	λ (cm^−1^)	Vibrational mode	Assignments
lDM1 and jDM1	No peak	NA	NA
Quadrant-4	λ (cm^−1^)	Vibrational mode	Assignments
Control and aDM1	1631	80% C=O stretching, 10% N–H bending, 10% C–N stretching	Amide-I: parallel β- sheets (peptide, protein)
	1534	60% N–H bending, 40% C–N stretching	Amide II (proteins)
	1515	CH_2_ bending	Lipid, protein
**1200–900 cm^−1^ Region**			
**Discrimination across PC-4**	**λ (cm^−1^)**	**Vibrational Mode**	**Assignments**
PC-4 − Control, lDM1, aDM1, iDM1	1155	C–O stretching, C–O–H bending	Carbohydrates
	1076	PO^2−^ symmetrical stretching	DNA, RNA, phospholipid, phosphorylated protein
	1025	C–O stretching and C–OH bending	DNA and RNA, oligosaccharides, polysaccharides (e.g., glucose)
	923	C–N^+^–C stretching	DNA and RNA ribose-phosphate chain vibration of RNA
PC-4 + jDM1 and cDM1	1121	Phosphodiester groups of PO^2−^	RNA
	1084	PO^2−^ symmetrical stretching	DNA, RNA, phospholipid, phosphorylated protein
	1047	C–O stretching and C–OH bending	DNA and RNA, oligosaccharides, polysaccharides (e.g., glucose)
	994	C–O stretching	DNA and RNA ribose
	968	PO_3_^2−^ stretching	DNA and RNA ribose
	931	C–N^+^–C stretching	DNA and RNA ribose-phosphate chain vibration of RNA
	914	C–N^+^–C stretching	DNA and RNA ribose-phosphate chain vibration of RNA
**1200–900 cm^−1^ Region (Q1, Q2, Q3 and Q4)**			
**Quadrant-1**	**λ (cm^−1^)**	**Vibrational Mode**	**Assignments**
jDM1	1124	Phosphodiester groups of PO^2−^	RNA
	996	C–O stretching	DNA and RNA ribose
Quadrant-2	λ (cm^−1^)	Vibrational mode	Assignments
cDM1	914	C–N^+^–C stretching	DNA and RNA ribose-phosphate chain vibration of RNA
Quandrant-3	λ (cm^−1^)	Vibrational mode	Assignments
aDM1	1112	Phosphodiester groups of PO^2−^	RNA
Quadrant-4	λ (cm^−1^)	Vibrational mode	Assignments
Control, lDM1 and iDM1	1155	C–O stretching, C–O–H bending	Carbohydrates
	1079	PO^2−^ symmetrical stretching	DNA, RNA, phospholipid, phosphorylated protein
	1042	C–O stretching and C–OH bending	DNA and RNA, oligosaccharides, polysaccharides (e.g., glucose)
	1022		
	926	C–N^+^–C stretching	DNA and RNA ribose-phosphate chain vibration of RNA

## Data Availability

Not applicable.

## Supplementary Materials

The following are available online at https://www.mdpi.com/article/10.3390/ijerph18073800/s1, Figure S1. FTIR spectra of DM1-derived fibroblasts and control from Coriell Institute. (A) Baseline-corrected and area normalized spectra of DM1-derived fibroblasts and control from Coriell Institute in the 4000–600 cm^−1^ region. (B) Amplification of the region 3000–2800 cm^−1^, (C) amplification of the region 1800–1500 cm^−1^, (D) amplification of the 1200–900 cm^−1^ region [21,26,30,34,36]. *X*-axis- wavenumber cm^−1^, *Y*-axis- arbitrary units (A.U.). Figure S2. Loading profile of DM1-derived fibroblasts and control from Coriell Institute. (A) 3000–2800 cm^−1^ region, (B) 1800–1500 cm^−1^ region, (C) 1200–900 cm^−1^ region. Figure S3. PCA scores of Coriell Institute and Neurolab UA DM1-derived fibroblasts and controls. (A) 3000–2800 cm^−1^ region, (B) 1800–1500 cm^−1^ region, (C) 1200–900 cm^−1^ region. Figure S4. FTIR spectra of DM1-derived fibroblasts and control cultured at Neurolab. (A) Baseline-corrected and area normalized spectra of DM1-derived fibroblasts and control from Neurolab UA in the 4000–600 cm^−1^ region. (B) Amplification of the region 3000–2800 cm^−1^, (C) amplification of the region 1800–1500 cm^−1^, (D) amplification of the 1200–900 cm^−1^ region [21,26,30,34,36]. *X*-axis- wavenumber cm^−1^, *Y*-axis- arbitrary units (A.U.). Figure S5. Loading profile of DM1-derived fibroblasts and control cultured at Neurolab. (A) 3000–2800 cm^−1^ region, (B) 1800–1500 cm^−1^ region, (C) 1200–900 cm^−1^ region.

## References

[1] Johnson N.E. (2019). Myotonic Muscular Dystrophies. Contin. Lifelong Learn. Neurol..

[2] Bozovic I., Peric S., Pesovic J., Bjelica B., Brkusanin M., Basta I., Bozic M., Sencanic I., Marjanovic A., Brankovic M. (2018). Myotonic Dystrophy Type 2 – Data from the Serbian Registry. J. Neuromuscul. Dis..

[3] Vanacore N., Rastelli E., Antonini G., Bianchi M.L.E., Botta A., Bucci E., Casali C., Costanzi-Porrini S., Giacanelli M., Gibellini M. (2016). An Age-Standardized Prevalence Estimate and a Sex and Age Distribution of Myotonic Dystrophy Types 1 and 2 in the Rome Province, Italy. Neuroepidemiology.

[4] Rodríguez R., Hernández-Hernández O., Magaña J.J., González-Ramírez R., García-López E.S., Cisneros B. (2015). Altered nuclear structure in myotonic dystrophy type 1-derived fibroblasts. Mol. Biol. Rep..

[5] Esposito F., Cè E., Rampichini S., Monti E., Limonta E., Fossati B., Meola G. (2017). Electromechanical delays during a fatiguing exercise and recovery in patients with myotonic dystrophy type 1. Eur. J. Appl. Physiol..

[6] Magaña J.J., Suárez-Sánchez R., Leyva-García N., Cisneros B., Hernández-Hernández O. (2012). Myotonic Dystrophy Protein Kinase: Structure, Function and Its Possible Role in the Pathogenesis of Myotonic Dystrophy Type 1. Advances in Protein Kinases.

[7] Cho D.H., Tapscott S.J. (2007). Myotonic dystrophy: Emerging mechanisms for DM1 and DM2. Biochim. Biophys. Acta Mol. Basis Dis..

[8] Thornton C.A. (2014). Myotonic Dystrophy. Neurol. Clin..

[9] De Antonio M., Dogan C., Hamroun D., Mati M., Zerrouki S., Eymard B., Katsahian S., Bassez G. (2016). Unravelling the myotonic dystrophy type 1 clinical spectrum: A systematic registry-based study with implications for disease classification. Rev. Neurol. (Paris)..

[10] Vujnic M., Peric S., Popovic S., Raseta N., Ralic V., Dobricic V., Novakovic I., Rakocevic-Stojanovic V. (2015). Metabolic syndrome in patients with myotonic dystrophy type 1. Muscle Nerve.

[11] Mateus T., Martins F., Nunes A., Herdeiro M.T. (2021). Metabolic Alterations in Myotonic Dystrophy Type 1 and Their Correlation with Lipin. Int. J. Environ. Res. Public Health.

[12] Ben Hamou A., Espiard S., Do Cao C., Ladsous M., Loyer C., Moerman A., Boury S., Kyheng M., Dhaenens C.-M., Tiffreau V. (2019). Systematic thyroid screening in myotonic dystrophy: Link between thyroid volume and insulin resistance. Orphanet J. Rare Dis..

[13] Renna L.V., Bosè F., Brigonzi E., Fossati B., Meola G., Cardani R. (2019). Aberrant insulin receptor expression is associated with insulin resistance and skeletal muscle atrophy in myotonic dystrophies. PLoS ONE.

[14] Renna L.V., Bosè F., Iachettini S., Fossati B., Saraceno L., Milani V., Colombo R., Meola G., Cardani R. (2017). Receptor and post-receptor abnormalities contribute to insulin resistance in myotonic dystrophy type 1 and type 2 skeletal muscle. PLoS ONE.

[15] Daniele A., De Rosa A., De Cristofaro M., Monaco M.L., Masullo M., Porcile C., Capasso M., Tedeschi G., Oriani G., Di Costanzo A. (2011). Decreased concentration of adiponectin together with a selective reduction of its high molecular weight oligomers is involved in metabolic complications of myotonic dystrophy type 1. Eur. J. Endocrinol..

[16] Shieh K., Gilchrist J.M., Promrat K. (2010). Frequency and predictors of nonalcoholic fatty liver disease in myotonic dystrophy. Muscle Nerve.

[17] Johansson A., Olsson T., Cederquist K., Forsberg H., Holst J., Ahren B. (2002). Abnormal release of incretins and cortisol after oral glucose in subjects with insulin-resistant myotonic dystrophy. Eur. J. Endocrinol..

[18] Johansson A., Boman K., Cederquist K., Forsberg H., Olsson T. (2001). Increased levels of tPA antigen and tPA/PAI-1 complex in myotonic dystrophy. J. Intern. Med..

[19] Cacciatore S., Loda M. (2015). Innovation in metabolomics to improve personalized healthcare. Ann. N. Y. Acad. Sci..

[20] Ellis D.I., Dunn W.B., Griffin J.L., Allwood J.W., Goodacre R. (2007). Metabolic fingerprinting as a diagnostic tool. Pharmacogenomics.

[21] Derenne A., Vandersleyen O., Goormaghtigh E. (2014). Lipid quantification method using FTIR spectroscopy applied on cancer cell extracts. Biochim. Biophys. Acta Mol. Cell Biol. Lipids.

[22] Stuart B.H. (2012). Infrared Spectroscopy of Biological Applications: An Overview. Encyclopedia of Analytical Chemistry.

[23] Bujok J., Gąsior-Głogowska M., Marszałek M., Trochanowska-Pauk N., Zigo F., Pavľak A., Komorowska M., Walski T. (2019). Applicability of FTIR-ATR Method to Measure Carbonyls in Blood Plasma after Physical and Mental Stress. Biomed Res. Int..

[24] Stuart B.H. (2005). Infrared Spectroscopy: Fundamentals and Applications.

[25] Movasaghi Z., Rehman S., ur Rehman D.I. (2008). Fourier Transform Infrared (FTIR) Spectroscopy of Biological Tissues. Appl. Spectrosc. Rev..

[26] Lopes J., Correia M., Martins I., Henriques A.G., Delgadillo I., Da Cruz E Silva O., Nunes A. (2016). FTIR and Raman Spectroscopy Applied to Dementia Diagnosis Through Analysis of Biological Fluids. J. Alzheimer’s Dis..

[27] Oleszko A., Hartwich J., Wójtowicz A., Gąsior-Głogowska M., Huras H., Komorowska M. (2017). Comparison of FTIR-ATR and Raman spectroscopy in determination of VLDL triglycerides in blood serum with PLS regression. Spectrochim. Acta Part A Mol. Biomol. Spectrosc..

[28] Wang L., Mizaikoff B. (2008). Application of multivariate data-analysis techniques to biomedical diagnostics based on mid-infrared spectroscopy. Anal. Bioanal. Chem..

[29] Santos F., Magalhães S., Henriques M.C., Silva B., Valença I., Ribeiro D., Fardilha M., Nunes A. (2019). Understanding Prostate Cancer Cells Metabolome: A Spectroscopic Approach. Curr. Metabolomics.

[30] Igci N., Sharafi P., Ozel Demiralp D., Demiralp C., Yuce A., Dokmeci (Emre) S. (2017). Application of Fourier transform infrared spectroscopy to biomolecular profiling of cultured fibroblast cells from Gaucher disease patients: A preliminary investigation. Adv. Clin. Exp. Med..

[31] Pereira C.D., Martins F., Santos M., Müeller T., da Cruz e Silva O.A.B., Rebelo S. (2020). Nuclear Accumulation of LAP1:TRF2 Complex during DNA Damage Response Uncovers a Novel Role for LAP1. Cells.

[32] Martins F., Serrano J.B., Müller T., da Cruz e Silva O.A.B., Rebelo S. (2017). BRI2 Processing and Its Neuritogenic Role Are Modulated by Protein Phosphatase 1 Complexing. J. Cell. Biochem..

[33] Staniszewska-Slezak E., Wiercigroch E., Fedorowicz A., Buczek E., Mateuszuk L., Baranska M., Chlopicki S., Malek K. (2018). A possible Fourier transform infrared-based plasma fingerprint of angiotensin-converting enzyme inhibitor-induced reversal of endothelial dysfunction in diabetic mice. J. Biophotonics.

[34] Felgueiras J., Silva J.V., Nunes A., Fernandes I., Patrício A., Maia N., Pelech S., Fardilha M. (2020). Investigation of spectroscopic and proteomic alterations underlying prostate carcinogenesis. J. Proteomics.

[35] Yonar D., Ocek L., Tiftikcioglu B.I., Zorlu Y., Severcan F. (2018). Relapsing-Remitting Multiple Sclerosis diagnosis from cerebrospinal fluids via Fourier transform infrared spectroscopy coupled with multivariate analysis. Sci. Rep..

[36] Oleszko A., Olsztyńska-Janus S., Walski T., Grzeszczuk-Kuć K., Bujok J., Gałecka K., Czerski A., Witkiewicz W., Komorowska M. (2015). Application of FTIR-ATR Spectroscopy to Determine the Extent of Lipid Peroxidation in Plasma during Haemodialysis. Biomed Res. Int..

[37] Du H., Cline M.S., Osborne R.J., Tuttle D.L., Clark T.A., Donohue J.P., Hall M.P., Shiue L., Swanson M.S., Thornton C.A. (2010). Aberrant alternative splicing and extracellular matrix gene expression in mouse models of myotonic dystrophy. Nat. Struct. Mol. Biol..

[38] Wang E.T., Treacy D., Eichinger K., Struck A., Estabrook J., Olafson H., Wang T.T., Bhatt K., Westbrook T., Sedehizadeh S. (2019). Transcriptome alterations in myotonic dystrophy skeletal muscle and heart. Hum. Mol. Genet..

[39] Chen Y., Rui B.-B., Tang L.-Y., Hu C.-M. (2015). Lipin Family Proteins - Key Regulators in Lipid Metabolism. Ann. Nutr. Metab..

[40] Okuno H., Okuzono H., Hayase A., Kumagai F., Tanii S., Hino N., Okada Y., Tachibana K., Doi T., Ishimoto K. (2019). Lipin-1 is a novel substrate of protein phosphatase PGAM5. Biochem. Biophys. Res. Commun..

[41] Péterfy M., Phan J., Reue K. (2005). Alternatively Spliced Lipin Isoforms Exhibit Distinct Expression Pattern, Subcellular Localization, and Role in Adipogenesis. J. Biol. Chem..

[42] Finck B.N., Gropler M.C., Chen Z., Leone T.C., Croce M.A., Harris T.E., Lawrence J.C., Kelly D.P. (2006). Lipin 1 is an inducible amplifier of the hepatic PGC-1α/PPARα regulatory pathway. Cell Metab..

[43] Kumar S., Srinivasan A., Nikolajeff F. (2017). Role of Infrared Spectroscopy and Imaging in Cancer Diagnosis. Curr. Med. Chem..

[44] Perczel A., Gáspári Z., Csizmadia I.G. (2005). Structure and stability of β-pleated sheets. J. Comput. Chem..

[45] Wheeler T.M., Krym M.C., Thornton C.A. (2007). Ribonuclear foci at the neuromuscular junction in myotonic dystrophy type 1. Neuromuscul. Disord..

[46] Ravel-Chapuis A., Bélanger G., Yadava R.S., Mahadevan M.S., DesGroseillers L., Côté J., Jasmin B.J. (2012). The RNA-binding protein Staufen1 is increased in DM1 skeletal muscle and promotes alternative pre-mRNA splicing. J. Cell Biol..

[47] Ramana K.V., Srivastava S., Singhal S.S. (2013). Lipid Peroxidation Products in Human Health and Disease. Oxid. Med. Cell. Longev..

[48] Wills E.D. (1966). Mechanisms of lipid peroxide formation in animal tissues. Biochem. J..

[49] Ayala A., Muñoz M.F., Argüelles S. (2014). Lipid peroxidation: Production, metabolism, and signaling mechanisms of malondialdehyde and 4-hydroxy-2-nonenal. Oxid. Med. Cell. Longev..

