# Supplementary material for: Fourier-Transform Infrared Spectroscopy as a Discriminatory Tool for Myotonic Dystrophy Type 1 Metabolism: A Pilot Study
Source: Int J Environ Res Public Health. 2021 Apr 6;18(7):3800. doi: 10.3390/ijerph18073800 (PMC8038712; doi:10.3390/ijerph18073800)
Supplement: Supplementary file 1 [file ijerph-18-03800-s001.pdf]

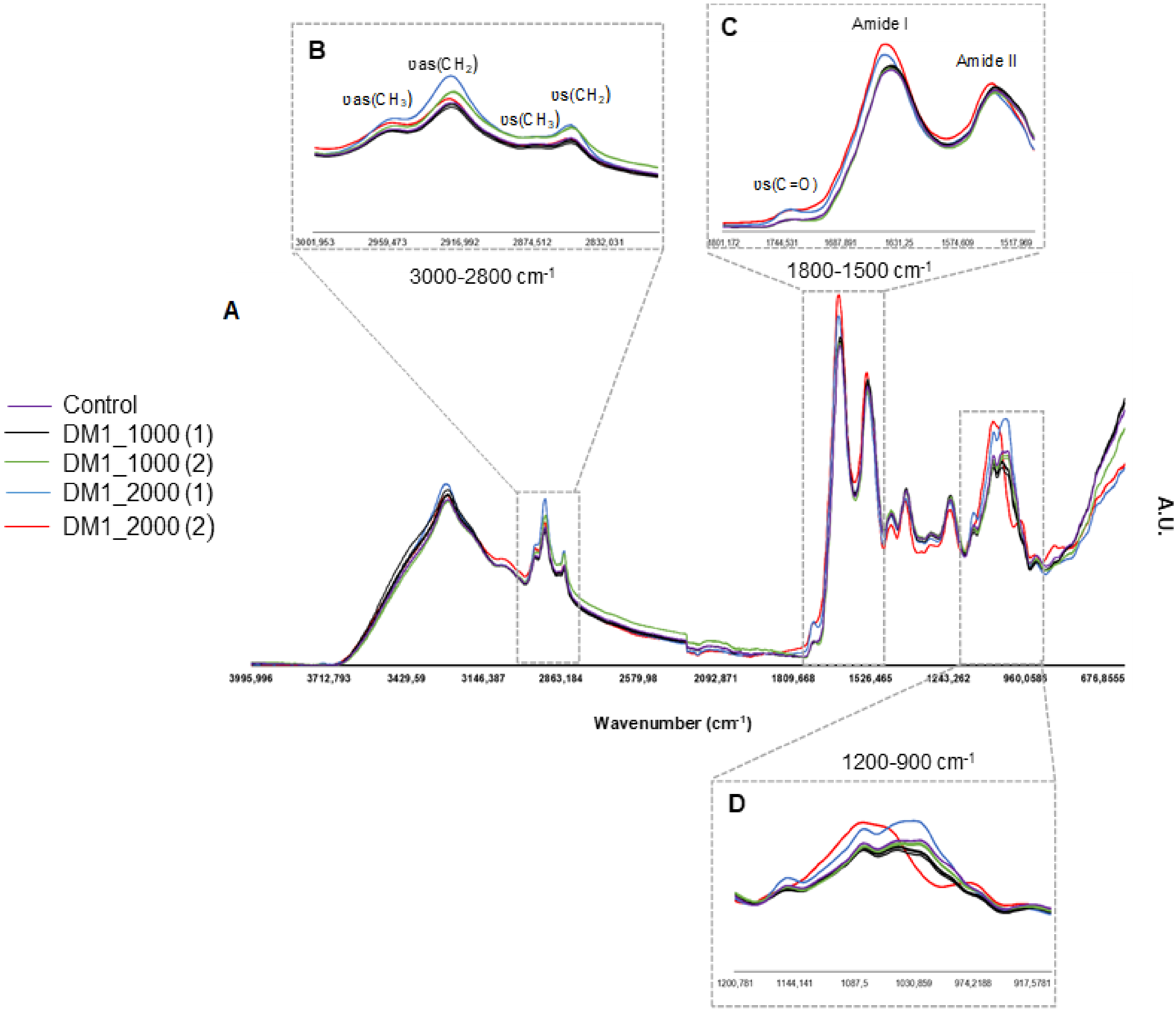

Figure S1. FTIR spectra of DM1-derived fibroblasts and control from Coriell Institute.

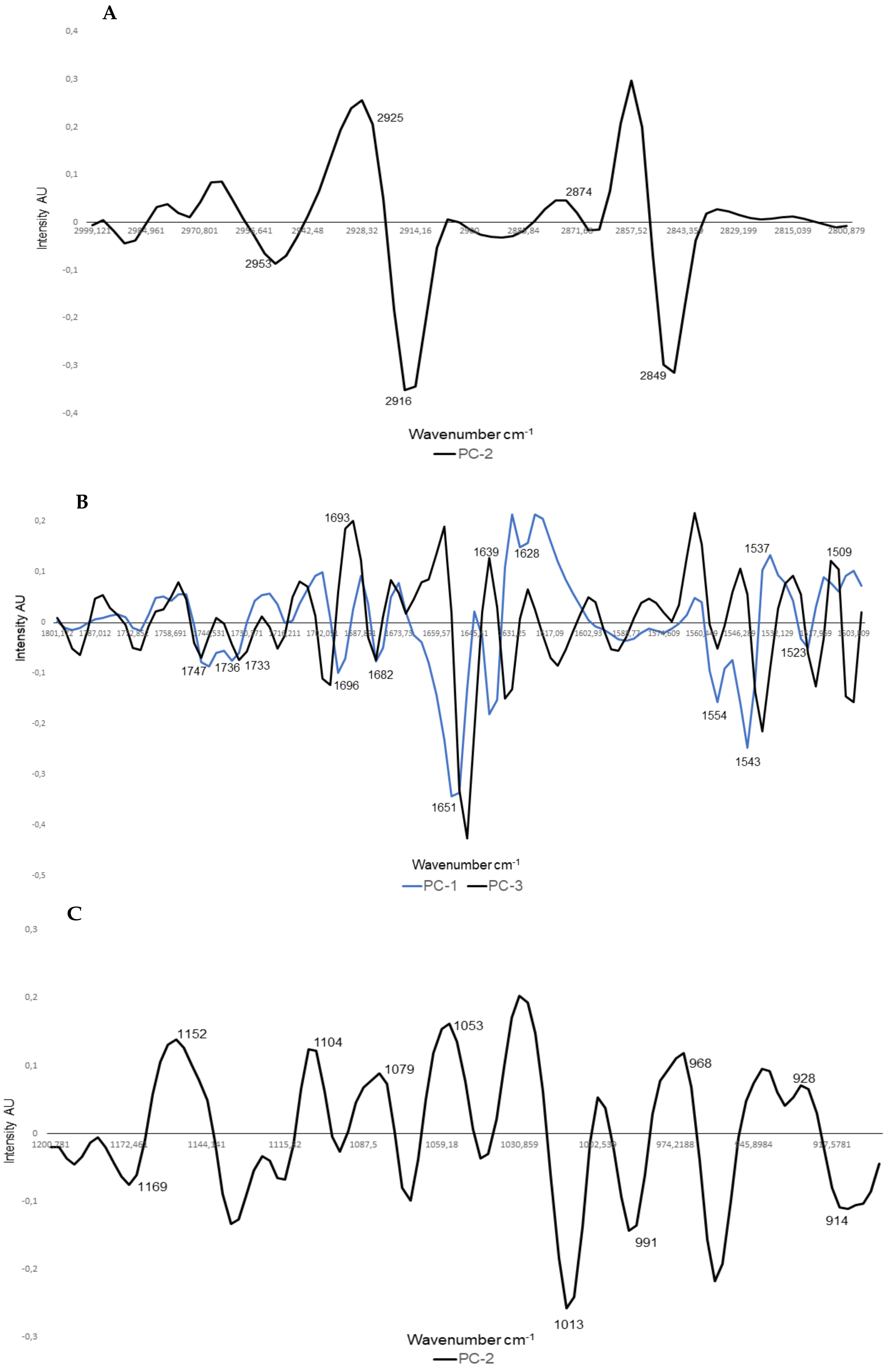

Figure S2. Loading profile of DM1-derived fibroblasts and control from Coriell Institute.

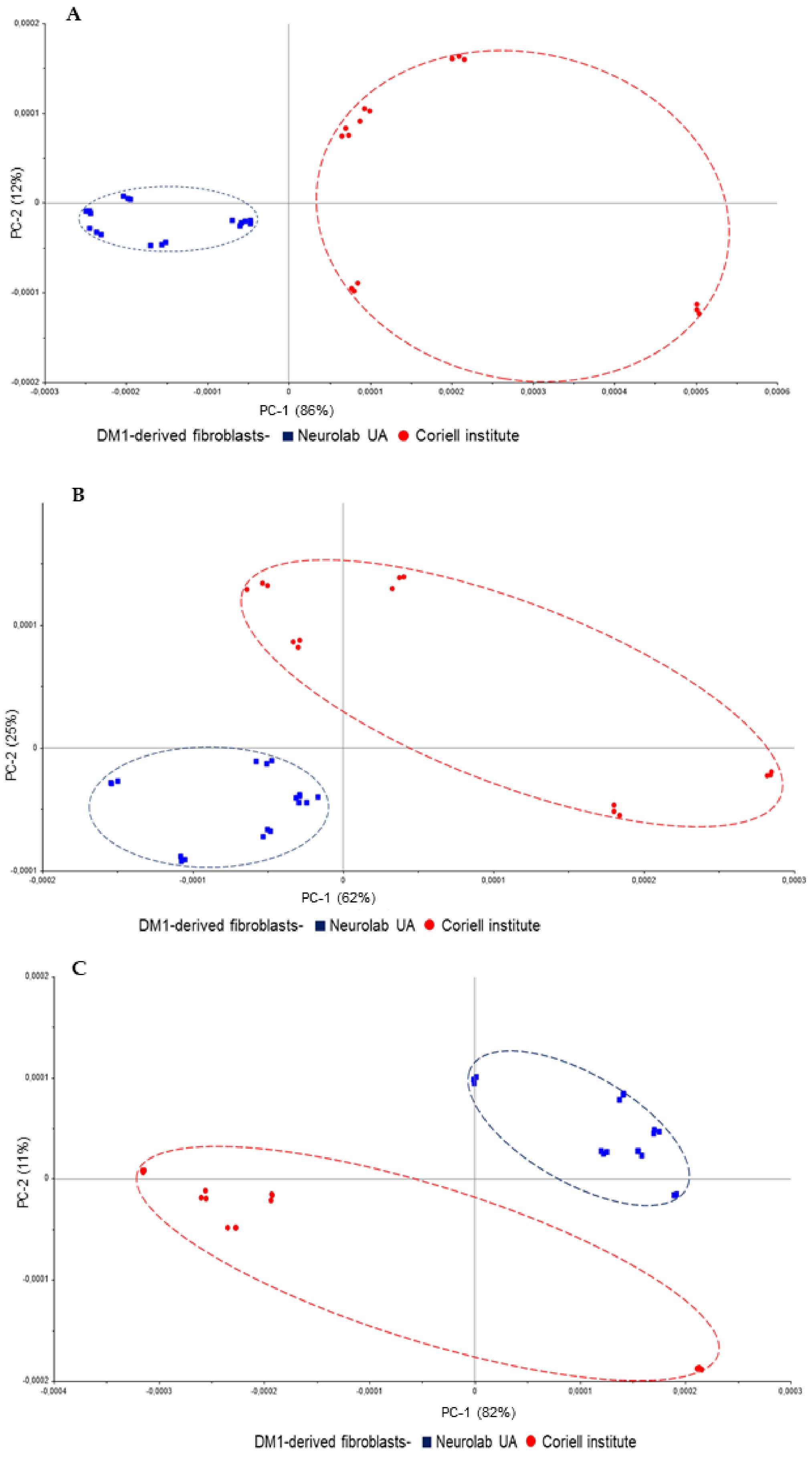

Figure S3. PCA scores of Coriell Institute and Neurolab UA DM1-derived fibroblasts and controls.

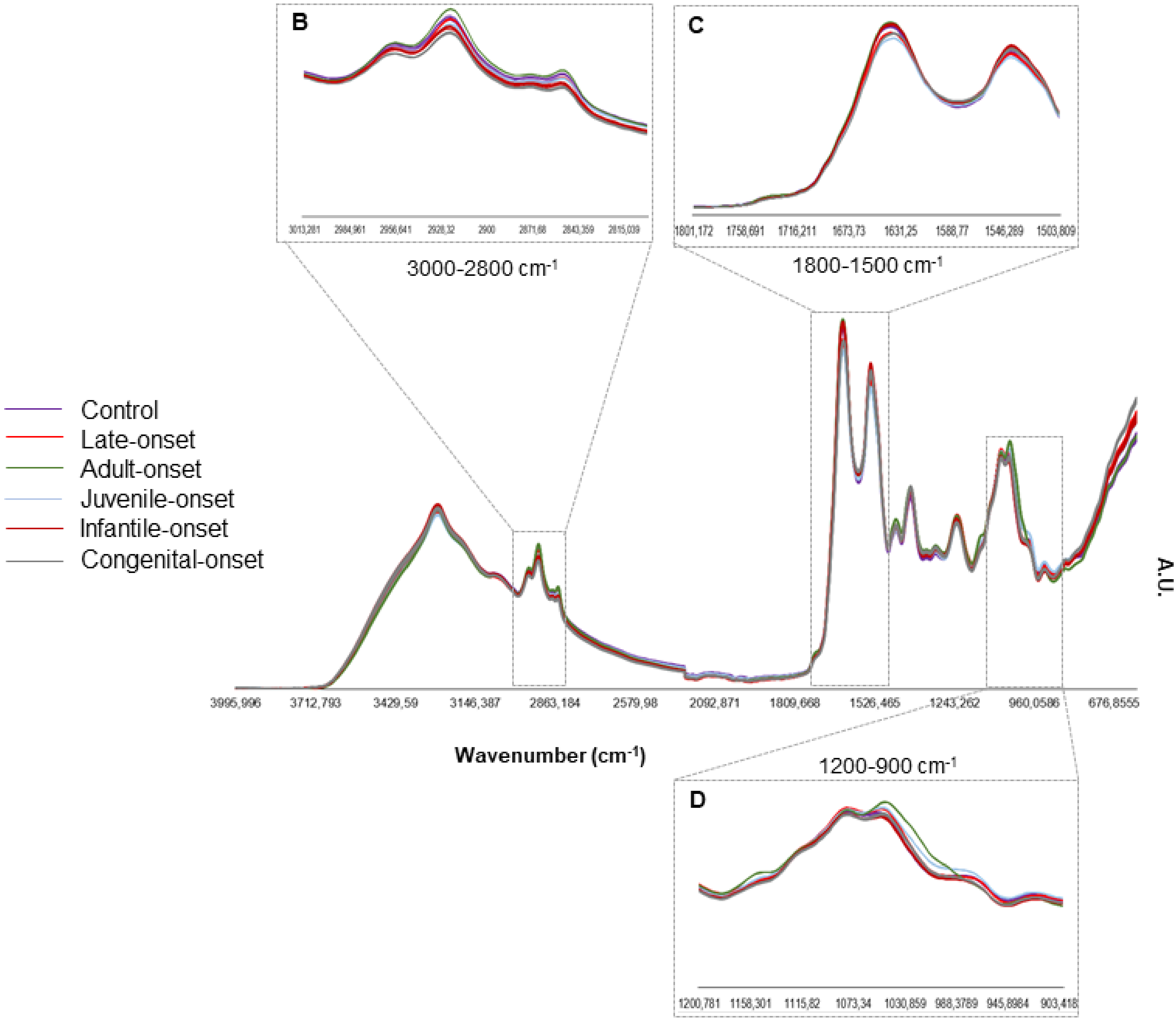

Figure S4. FTIR spectra of DM1-derived fibroblasts and control cultured at Neurolab.

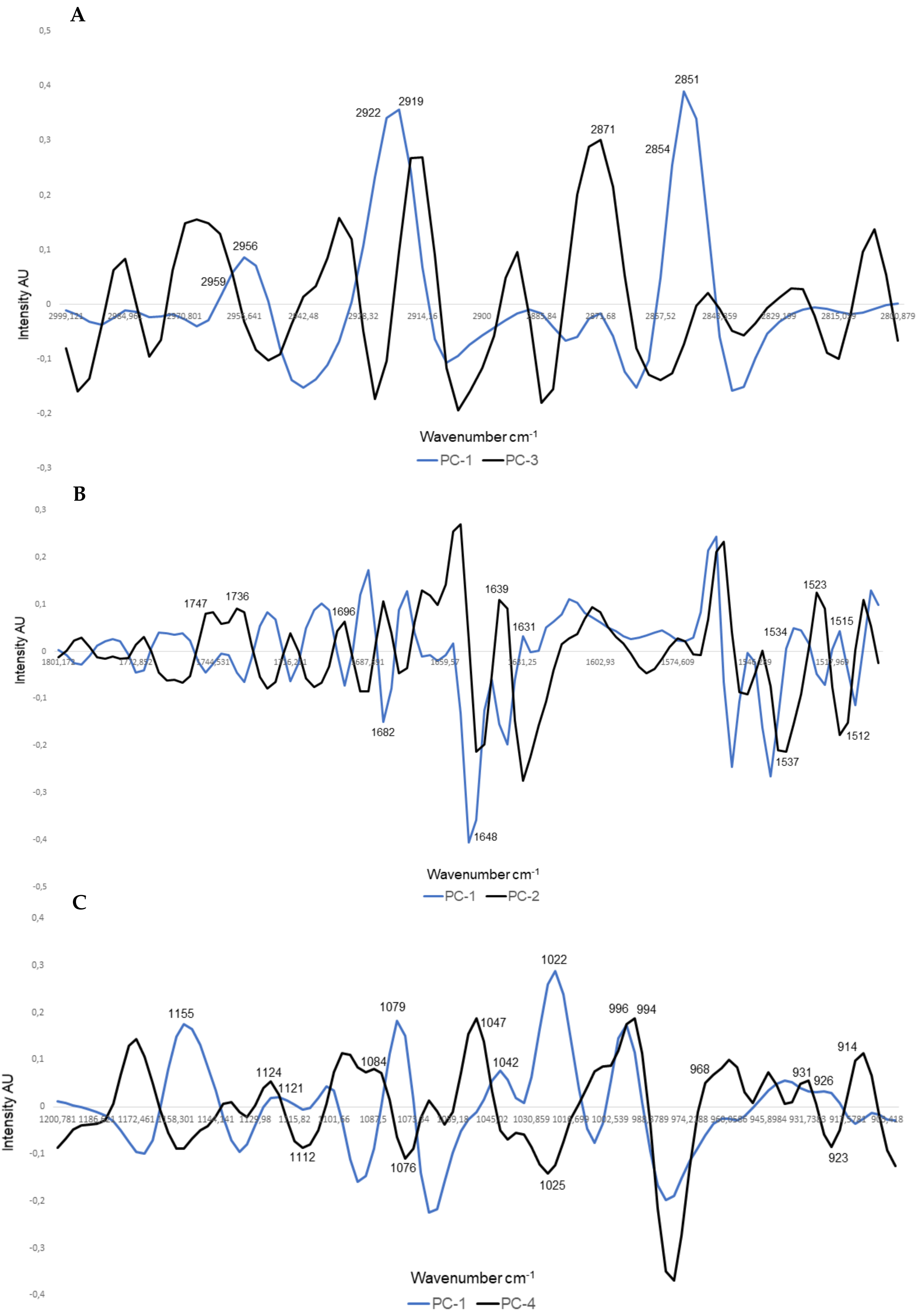

Figure S5. Loading profile of DM1-derived fibroblasts and control cultured at Neurolab.
